# Three-dimensional nanoframes with dual rims as nanoprobes for biosensing

**DOI:** 10.1038/s41467-022-32549-w

**Published:** 2022-08-16

**Authors:** Hajir Hilal, Qiang Zhao, Jeongwon Kim, Sungwoo Lee, MohammadNavid Haddadnezhad, Sungjae Yoo, Soohyun Lee, Woongkyu Park, Woocheol Park, Jaewon Lee, Joong Wook Lee, Insub Jung, Sungho Park

**Affiliations:** 1 Department of Chemistry, Sungkyunkwan University (SKKU), Suwon, 16419 Republic of Korea; 2 Medical & Bio Photonics Research Center, Korea Photonics Technology Institute (KOPTI), Gwangju, 61007 Republic of Korea; 3 Department of Physics and Optoelectronics Convergence Research Center, Chonnam National University, Gwangju, 61186 Republic of Korea; 4 Department of Chemistry and Institute of Basic Science, Sungkyunkwan University (SKKU), Suwon, 16419 Republic of Korea

**Keywords:** Nanoparticles, Synthesis and processing, SERS

## Abstract

Three-dimensional (3D) nanoframe structures are very appealing because their inner voids and ridges interact efficiently with light and analytes, allowing for effective optical-based sensing. However, the realization of complex nanoframe architecture with high yield is challenging because the systematic design of such a complicated nanostructure lacks an appropriate synthesis protocol. Here, we show the synthesis method for complex 3D nanoframes wherein two-dimensional (2D) dual-rim nanostructures are engraved on each facet of octahedral nanoframes. The synthetic scheme proceeds through multiple executable on-demand steps. With Au octahedral nanoparticles as a sacrificial template, sequential processes of edge-selective Pt deposition and inner Au etching lead to Pt octahedral mono-rim nanoframes. Then, adlayers of Au are grown on Pt skeletons via the Frank-van der Merwe mode, forming sharp and well-developed edges. Next, Pt selective deposition on both the inner and outer boundaries leads to tunable geometric patterning on Au. Finally, after the selective etching of Au, Pt octahedral dual-rim nanoframes with highly homogeneous size and shape are achieved. In order to endow plasmonic features, Au is coated around Pt frames while retaining their geometric shape. The resultant plasmonic dual-rim engraved nanoframes possess strong light entrapping capability verified by single-particle surface-enhanced Raman scattering (SERS) and show the potential of nanoprobes for biosensing through SERS-based immunoassay.

## Abstract (web summary)

Most SERS-active nanostructures suffer from low robustness against misalignment to field polarization. Here, the authors demonstrate three-dimensional nanoframes of octahedral geometry, with two rims engraved on each facet, as polarization-independent SERS nanoprobes.

## Introduction

Designing nanocrystals with structural complexity has long been a primary interest in nanoscience because their diverse physical and chemical properties can be greatly maximized^1–8^. Among diverse shapes such as spheres, rods, and stars, nanoframes (NFs) have shown great potential in many applications including sensing and imaging because each ridge and inner void allows strong light-matter interactions and their high exposed surface areas endow effective analyte sensing^9–14^. However, the reported synthesis of these NFs has been limited to three-dimensional (3D) mono-rim NFs where their structural functionality are not fully utilized^4^. The realization of more complex NFs with a high structural hierarchy is challenging. On the other hand, for the synthesis of plasmonic NFs, galvanic replacement reactions or lithography methods have been frequently adopted^13,15–19^. However, through the galvanic replacement reaction, systematically controlling the geometrical parameters of NFs (i.e., rim size) is difficult because the oxidation and reduction of noble metal ions occur simultaneously. In addition, through a top-down lithographical method, although it is feasible to produce nanostructures with high fidelity as well as high precision, controlling the physical dimensions of NFs in a 3D fashion is restricted. Furthermore, the construction of such a complex nanostructure in a bottom-up approach is even more difficult because many experimental parameters and side reactions likely cause inhomogeneous size and shape.

Recently, we reported the controlled synthesis of two-dimensional (2D) nanorings with dual rims wherein an electromagnetic field could be effectively confined between the inner and outer rims^20^. Also, recently, we have reported the synthesis of 3D Au truncated octahedral (TOh) dual-rim NFs where truncated flat terraces are formed on (100) facets^21^. Although flat terraces facilitated close-packing into monolayer arrays and contributed to highly sensitive surface-enhanced Raman scattering (SERS) signals when they were assembled as a bulk substrate, an individual particle showed polarization-dependent SERS activity and inferior intensity depending on the orientation, which hinders the generation of uniform SERS signals. In this context, if stereoscopic NFs can be produced with dual rims on every facet (i.e., dual-rims are all present on every facet), it would significantly amplify the interaction with light due to a gap-induced coupling in the 3D nanostructures. Additionally, controlling the intragap distance would be ideal to fine-tune the light trapping capability within such a complex nanostructure. Here, we demonstrate a synthesis strategy for highly advanced and intricate metallic 3D NF structures utilizing wet chemistry with multiple chemical reactions. The NFs are comprised of a 2D dual rim engraved in a 3D fashion with Pt skeletons, producing multiple intra-nanogaps with highly homogeneous size and shape. After a plasmonic component (i.e., Au) is coated, the near-field focusing capability is effectively enhanced due to multiple intra-nanogaps in a single entity, which enables single-particle SERS. The final structure is equivalent to 2D dual-rim patterns engraved in each facet of octahedral NPs in a 3D configuration, resulting in all frame-frame structures, which has never been reported. Importantly, we demonstrate that SERS-based immunoassay toward human chorionic gonadotropin (HCG) and that 3D Au dual-rim NFs show a higher detection sensitivity than that of 2D triangular counterparts, providing the potential use of 3D complex nanoframes architectures.

## Results and discussion

### Multiple chemical steps for the synthesis of Pt dual-rim NFs

**Fig. 1:**
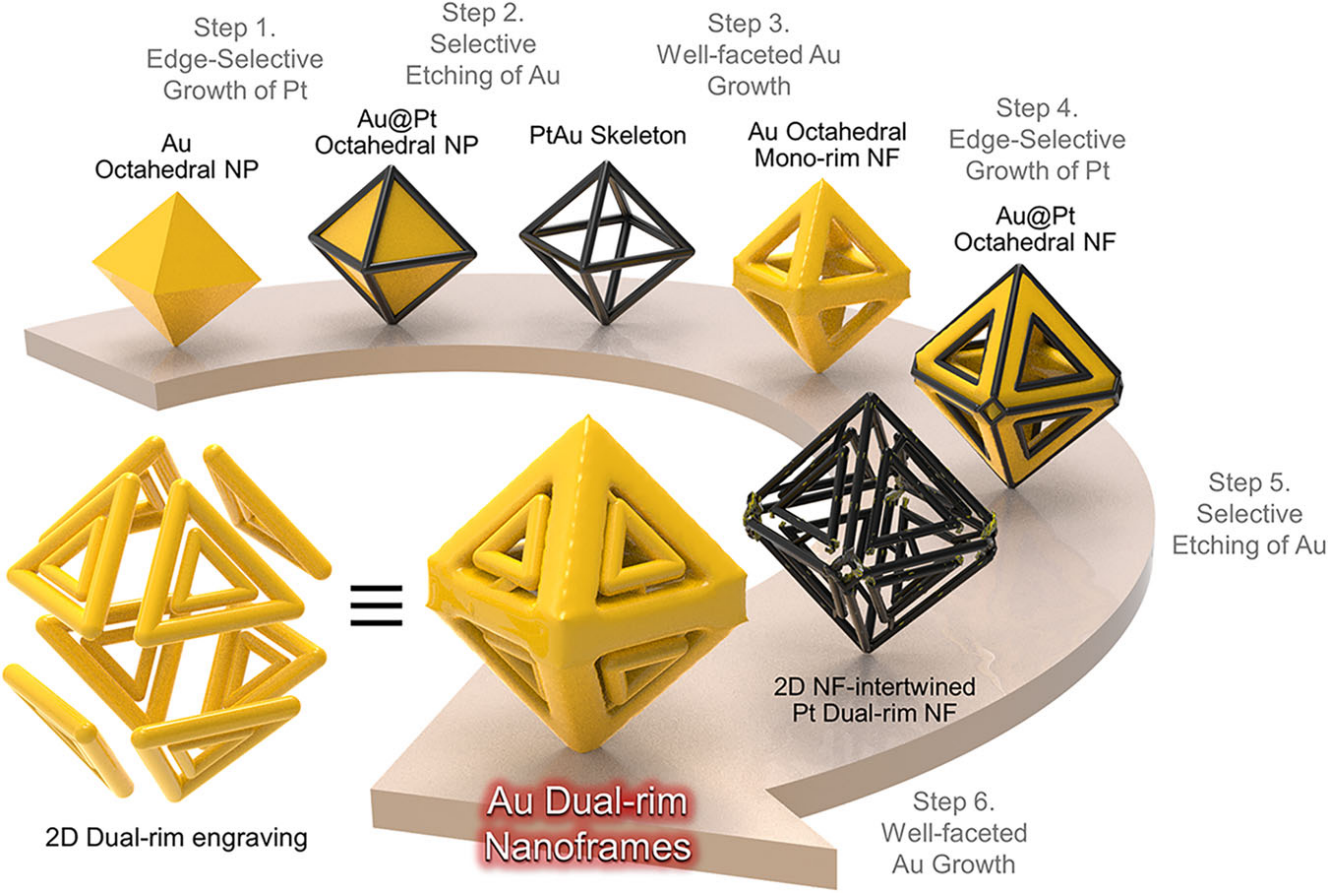
Schematic illustration of the synthesis strategy using multistep chemical reaction conditions. Multiple synthetic pathways for 2D nanoframes (NFs)-engraved octahedral 3D NFs by stepwise chemical toolkits.

**Fig. 2:**
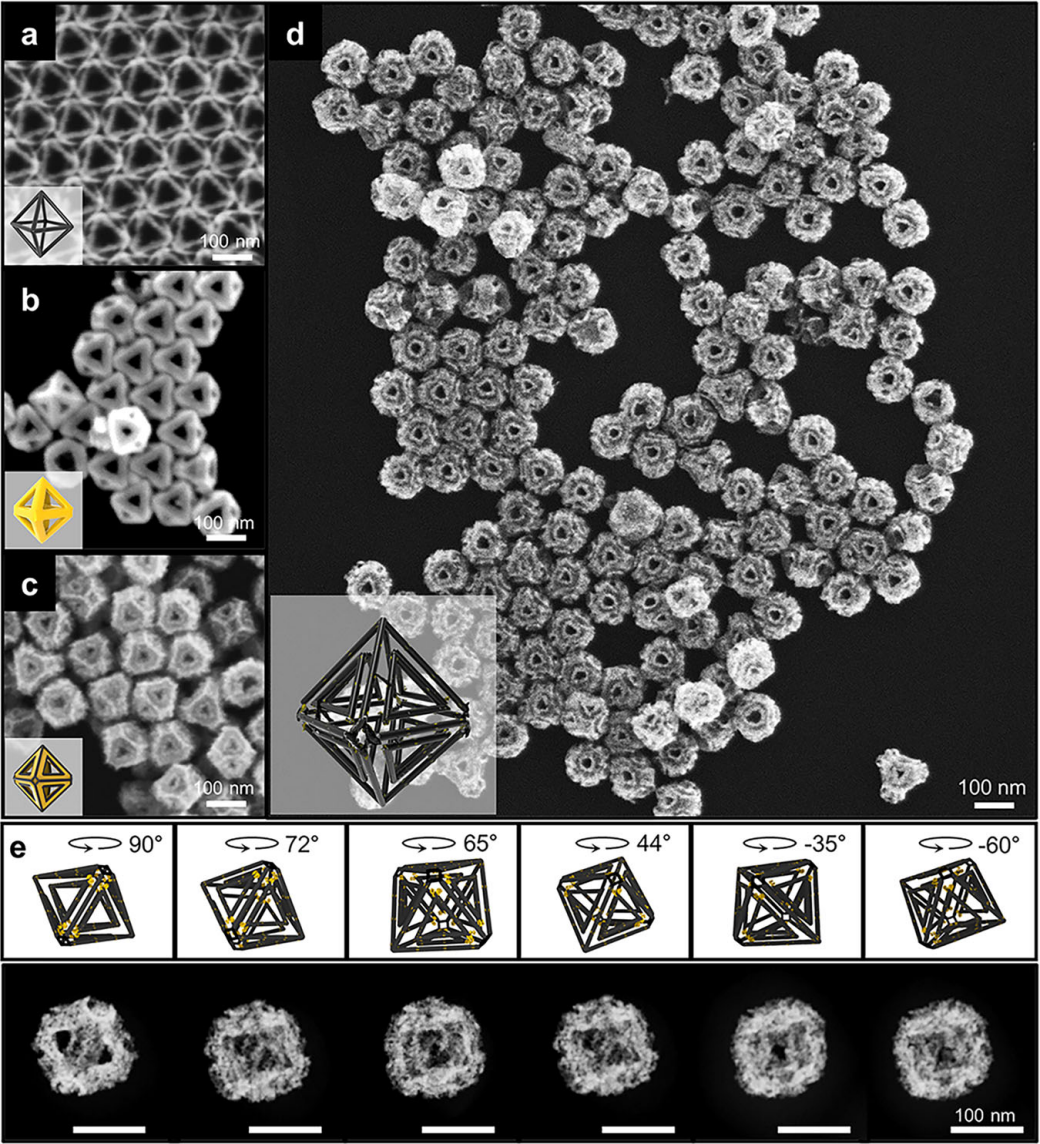
Morphological evolution for octahedral dual-rim NFs using multistep chemical reaction conditions. FE-SEM images of **a** Pt mono-rim skeletons, **b** Au mono-rim NFs, **c** Au@Pt NFs, and **d** Pt octahedral dual-rim NFs. **e** Rotational snapshots and cartoons of Pt dual-rim NFs at different angles of −60°, −35°, 44°, 65°, 72°, and 90°.

We adopted multiple stepwise chemical toolkits to achieve dual-rim NFs with highly homogeneous size and shape via wet chemistry, as shown in the schematic illustration (Fig. 1). Au solid octahedral nanoparticles (ca. 120 nm in diameter) (NPs) that are comprised of (111) planes were utilized as starting sacrificial templates^22^. The synthesis procedure consists of a multistep process as follows: (1) edge-selective growth of Pt, (2) selective etching of Au, (3) well-faceted Au growth, (4) second edge-selective growth of Pt, (5) second selective etching of Au, and (6) well-faceted Au growth. In step (1), Pt is deposited selectively along the 12 edges and 6 vertexes of Au octahedral NPs (where the surface energy is higher than that of the flat terraces) with the assistance of Ag^+^ ions. The thin layer of Ag formed by a reductant (ascorbic acid) works as an electron shuttle and induces Pt reduction via a galvanic replacement between Ag and Pt^4+^ (Supplementary Fig. 1)^23,24^. Under a mild Au etching condition with Au^3+^ ions, the inner Au octahedral NPs are selectively etched through comproportionation of Au^3^^+^, leaving Pt octahedral NFs with a rim thickness of ~10 nm, as shown in the field-emission scanning electron microscopy (FE-SEM) image (step 2 corresponding to Fig. 2a). The key to the synthesis of dual frame-engraved 3D NFs is described in step 3, wherein well-faceted Au growth proceeds along the Pt mono-rim skeleton with well-defined sharp edges, forming enlarged Au octahedral NFs. With the presence of Ag^+^ and Cl^−^ ions, the Pt skeleton acts as nucleation sites and a pre-formed thin layer of Ag due to the underpotential deposition on Pt rims facilitates a conformal reduction of Au onto Pt surfaces following the Frank-van der Merwe mode (i.e., layer-by-layer growth) (Fig. 2b). Since the growth rate of Au is uniform in all directions, the center voids created on each octahedral facet resemble triangular-like shapes. In addition, the sharp edges with a low-coordination number allow preferential Pt deposition on both the inner and outer edges (step 4, Fig. 2c). Without Ag^+^ ions during the reaction, dull edges (i.e., tip-rounded) are formed (Supplementary Fig. 2) because Au deposition on Pt is not favorable^25^ due to lattice mismatch between Pt (0.3912 nm) and Au (0.4065 nm). A small amount of residual Au atoms (Au atoms intercalated at the inner vertex sites of Pt NFs after the inner Au etching) facilitated the Au growth with the aid of ascorbic acid and Au is reduced faster on the vertex sites than along the rims, forming Au tip-rounded NFs. We further examined the Au growth patterns by tuning the experimental parameters (different halide ions (Cl^−^, Br^−^ and I^−^), acidity of growth solution (pH 3, 7 and 10) and the ratio of Ag^+^ to Au^3+^ ([Ag^+^]/[Au^3+^] ratio = 0.05, 0.1 and 1)) and determined the optimum conditions wherein it shows that Ag^+^ and Cl^−^ ions were critical for the formation of sharp edges (Supplementary Figs. 3 & 4). Instead, when I^−^ or Br^−^ ions were used with the CTAB surfactant, Au NFs with smooth or rough surfaces were obtained regardless of growth pH. Whereas sharp edges (i.e., well-faceted growth) are formed with Cl^−^ ions under the acidic (pH = 3) growth solution condition where underpotential deposition (UPD) of Ag was facilitated, allowing for the formation of uniform and sharp edges^26^. This is attributed to the higher electrochemical reduction potential of Ag (E°[Ag/AgCl] = 0.2223 V compared to E°[Ag/AgBr] = 0.0713 V and E°[Ag/AgI] = −0.1522 V) under the acidic (pH = 3) growth solution condition. Only concentric growth pattern of Au was achieved under the neutral (pH = 7) or basic (pH = 10) conditions. In addition, as the [Ag^+^]/[Au^3+^] ratio increased from 0.05 to 1, the concentration of Ag^+^ became high enough to fully wrap around the Pt skeletons, resulting in successful formation of Ag UPD layer for the growth of Au NFs with sharp edges (Supplementary Fig. 4). Importantly, this additional overgrowth of Au with well-defined facets enables the second rim during preferential Pt deposition (step 4) where Pt is reduced along the inner and outer edges (total 36 edges), resulting in Pt@Au@Pt octahedral NFs, which is clearly visible by the bright lines shown in Fig. 2c. By applying the selective etching of inner Au NFs again, dual rim-engraved Pt NFs were successfully achieved with high structural robustness and homogeneous size and shape, as evident in Fig. 2d, which was not achievable from NFs with dull edges (Supplementary Fig. 5). It was observed that the inner and outer nanoframes were held by thin metal bridges, endowing the dual frame structures without losing the inner frames. It is noteworthy to mention that the homogeneity and yield of NFs are very high (> 95%), which indicates that the synthesis steps suppress new nucleation and exclusively proceed on the nanoparticle scaffold. To reveal the internal morphology of our complex 3D structure, we carried out electron tomography, as shown in Fig. 2e. The rotational snapshots of high-angle annular dark field-scanning transmission electron microscopy (HAADF-STEM) images taken at different angles of −60°, −35°, 44°, 65°, 72°, and 90° clearly demonstrate that the dual rims are well-patterned on each facet as designed (also see the Supplementary Movie 1).

### Morphological and optical characterization of Au dual-rim NFs

**Fig. 3:**
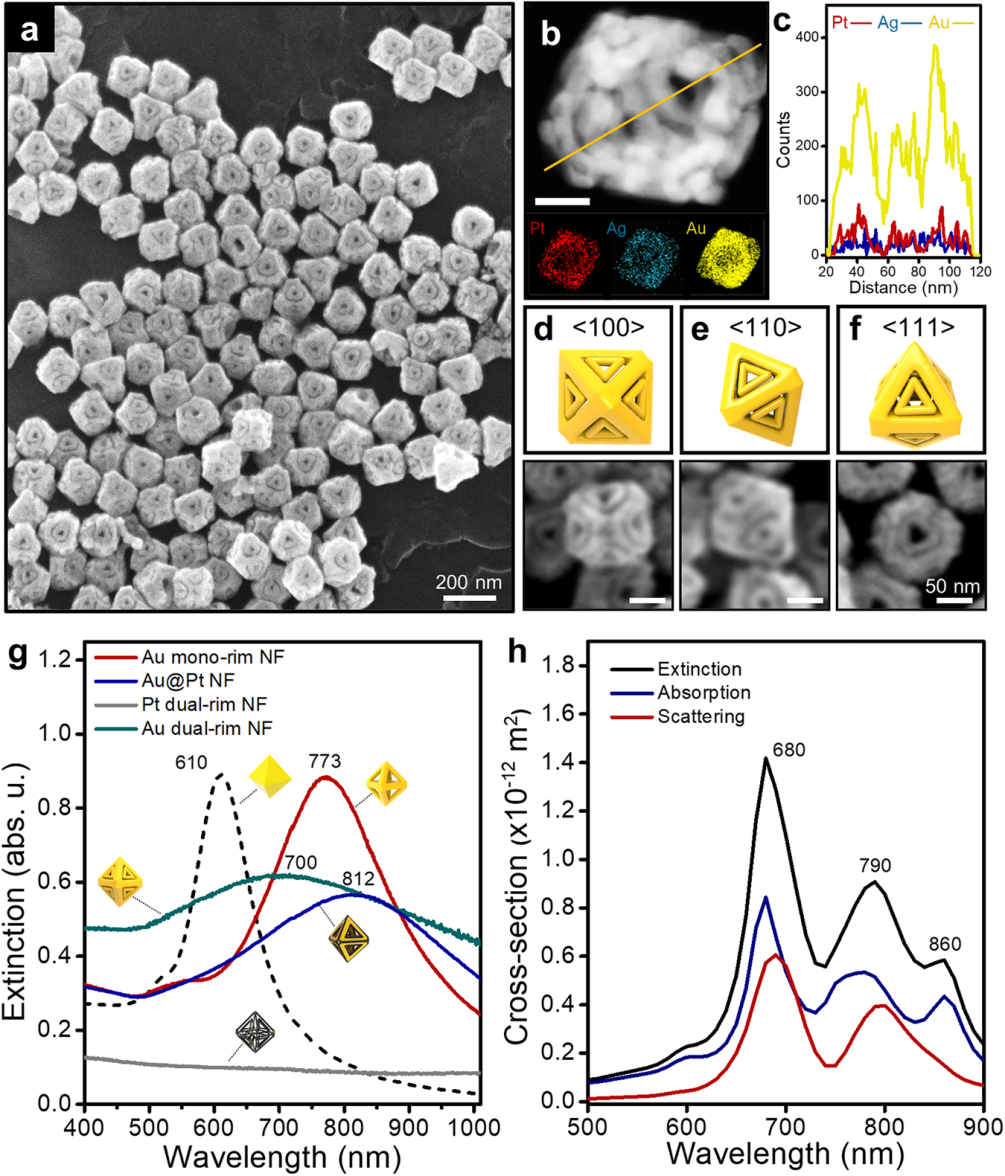
Structural characterization of Au octahedral dual-rim NFs. **a** A low-resolution FE-SEM image of dual rim-engraved Au NFs. **b** A HAADF-STEM image of Au dual-rim NFs, energy dispersive spectroscopy (EDS) elemental mapping, and **c** EDS line mapping for Au, Ag and Pt components. The scale bar denotes 50 nm. **d–f** FE-SEM images and cartoons viewed from <100>, <110>, and <111> directions, respectively. **g** Extinction spectra of Au mono-rim NFs, Au@Pt NFs, Pt dual-rim NFs, and Au dual-rim NFs. The numbers denote localized surface plasmon peaks for each particle. **h** Calculated average optical extinction, absorption, and scattering spectra of Au octahedral dual-rim NFs.

Since Pt dual-rim skeletons are structurally robust, secondary metal ions can be easily reductively coated without affecting the core Pt frameworks during multiple chemical reactions. Representatively, we deposited the plasmonic-active Au component by the reduction of Au^3+^, leading to dual rim-engraved 3D Au nanoframes. The low-resolution FE-SEM image shows very complex but distinct Au dual rim-engraved NFs with high homogeneity (Fig. 3a). In addition, the zoomed-in transmission electron microscopy (TEM) image taken at the <110> direction in addition to the energy-dispersive spectroscopy (EDS) and EDS line mapping analyses (Fig. 3b, c) further prove the structural complexity. The structure exhibits inner open spaces with all triangular dual rim-framed in the octahedron nanostructures with an atomic percentage of Ag (10.04%), Au (75.79%), and Pt (14.18%) (Supplementary Fig. 6). It is noticeable that the nanogaps between the dual rims are clearly represented in all octahedron faces, as shown in the FE-SEM images obtained from different angles (<100>, <110>, <111>) (Fig. 3d– f), while the structural complexity and intricacy of NFs are maintained during the multiple chemical steps. The Au dual-rim NFs are structurally equivalent to eight dual-rim triangular nanoplates connected in an octahedral configuration. The optical characterization of each morphological evolution by visible-near infrared (vis-NIR) spectroscopy is shown in Fig. 3g. Because Pt is plasmonically less active in the visible and NIR regions, Pt mono-rim NFs show a feature-less broad spectrum in the measured spectral window (Supplementary Fig. 1). A small amount of Au adatoms on the inner side of the Pt rims is not enough to produce localized surface plasmon resonance (LSPR). After the well-faceted growth of Au on Pt skeletons, a localized surface plasmon peak, a dipole mode centered at 773 nm, appears (red spectrum, Fig. 3g). After the selective rim upon Pt deposition, surface plasmon coupling between Au and Pt (i.e., behaves larger Au NFs) induces a redshift of the dipole mode to 812 nm with dampened intensity (blue spectrum, Fig. 3g) and the LSPR disappears after Au etching (gray spectrum, Fig. 3g), implying only Pt segments are left in the dual-rim NF structures. Finally, the concentric Au growth on Pt dual-rim NFs produces a LSPR of 700 nm (green spectrum, Fig. 3g). The calculated average absorption, scattering, and extinction profile of Au dual-rim NFs with a 5 nm gap are shown in Fig. 3h (see Supplementary Fig. 7 for the calculated optical profile of Au dual-rim NFs under different oscillating directions of the electric field). The dipole plasmon extinction peak is located at 680 nm with two additional peaks of 790 and 860 nm, which arise from the gap-induced plasmonic coupling^27^. Interestingly, the experimental ensemble results of the plasmonic band of dual-rim Au NFs are very broad, covering a wide range of visible and NIR regimes originating from the inhomogeneous size distribution of the real sample and the ensemble effect from the solution. In addition, a dark-field spectroscopy for individual Au dual-rim NFs was conducted, showing one dominant peak centered around 700 nm (Supplementary Fig. 8). We attributed this to highly complex architectures and multiple nanogaps in dual-rim NFs that give rise to the broadening of plasmon bands. It should be noted that the high complexity and fragility of Au dual-rim NFs under a high electron dose during the STEM measurement makes it hard to achieve 3D tomographic images through 3D reconstruction. Indeed, an investigation using FE-SEM images was more helpful in visualizing and analyzing the morphological architectures of our complex nanoparticles. Electron microscopy analysis for more in-depth morphological investigation of NFs will be addressed in the near future.

### Control of gap distances between the inner and outer rims of Pt dual-rim NFs

**Fig. 4:**
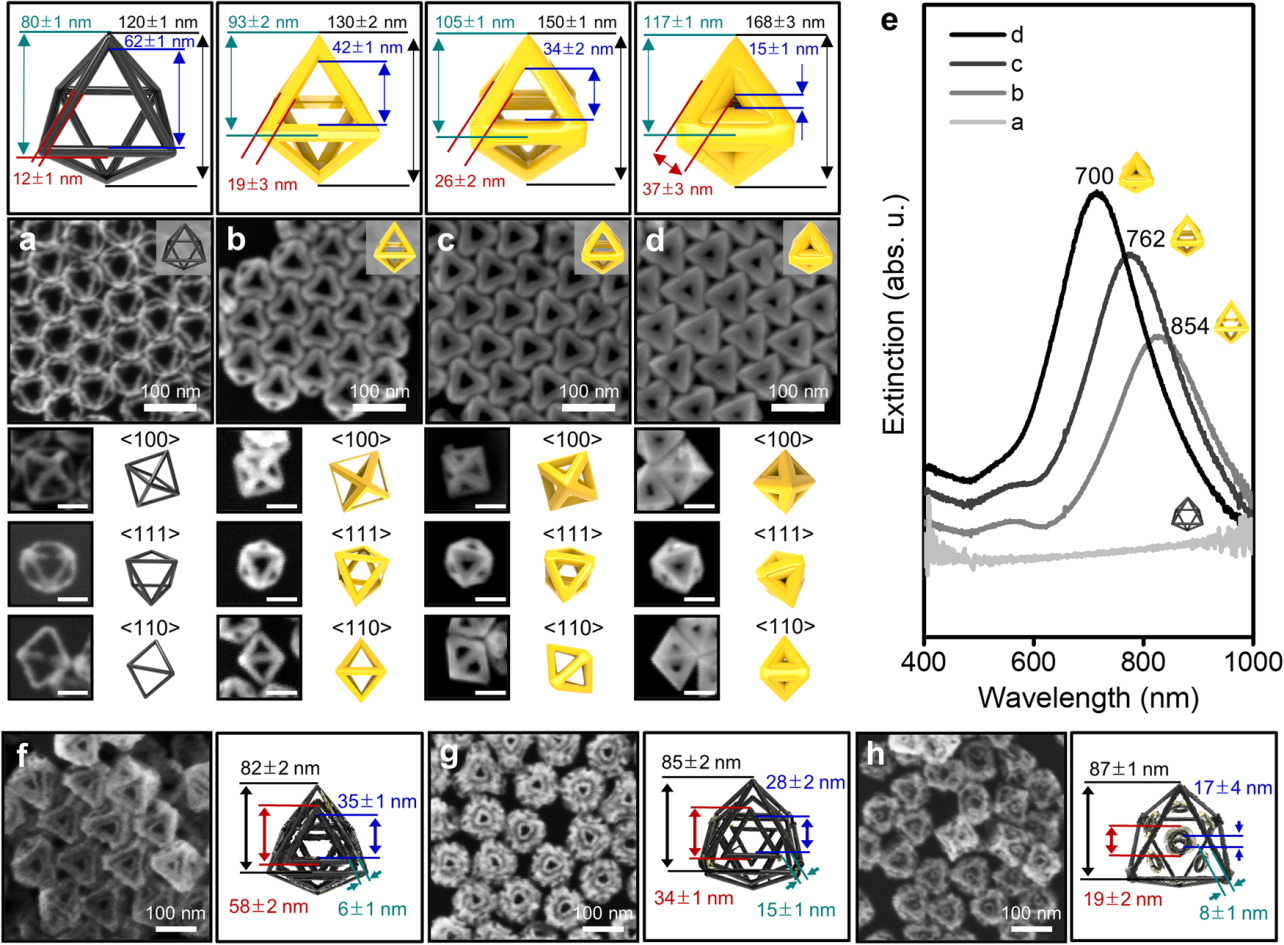
Gap controllability of well-faceted Au growth on PtAu octahedral skeletons and control of the gap distances between the inner and outer rims of Pt octahedral dual-rim NFs. **a**–**d** FE-SEM images of the growth evolution in the well-faceted growth step with the Pt skeleton as starting templates and cartoons describing dimensions of each particle measured from at least 30 nanoparticles. The FE-SEM images obtained at different angles demonstrate the high quality of both the size and shape of the as-prepared Au mono-rim NFs. **e** Corresponding UV-vis-NIR spectra with different gap sizes. **f–h** Pt octahedral dual-rim NFs with different nanogap sizes along with size information. The error bars represent standard deviations from 50 particles.

The stepwise synthesis protocol reported here allows one to fine-tune the gaps between the inner and outer rims of Au by controlling well-faceted growth that determines the thickness of the inner and outer rims of Pt dual-rim NFs. With Pt mono-rim skeletons with a rim size of 12 ± 1 nm and overall size of 120 ± 1 nm, we controlled the amount of Au ions while keeping the other parameters the same. Homogeneous Au growth can be clearly observed with increases of the overall size (from 130 ± 2, 150 ± 1 to 168 ± 3 nm) and rim thickness (from 19 ± 3, 26 ± 2 to 37 ± 3 nm), as shown in Fig. 4a–d (the size information of each NP is shown in the cartoons). It is worth mentioning that the sharp edges are obviously maintained during the growth. Corresponding UV-vis-NIR spectra are shown in Fig. 4e and the plasmonic bands continuous blue-shift from 854, 762 to 700 nm accompanying a decrease of the gap size from 42 ± 1, 34 ± 2 to 15 ± 1 nm. The Au rim thickness in the well-faceted growth step dictates not only the intra-nanogaps of Pt dual-rim NFs (Supplementary Fig. 9), but also the structural homogeneity of Pt NFs. When we synthesize Pt dual-rim NFs starting from Au mono-rim NFs with thin rims (e.g., Fig. 4b), the distances between the Pt inner and outer rims are too close to be clearly distinguished (Fig. 4f). Certain regions are merged together and others are separated from each other, although the overall shape retains the dual-rim NF morphology. If the rim is too thick (the case in Fig. 4d), the location of the inner Pt rims is not consistent, wherein the inner Pt triangular rims are tilted toward one direction (Fig. 4h). To synthesize Pt dual-rim NFs with clear and uniform intra-gaps as shown in Fig. 4g, appropriate thickness control in the well-faceted growth of the Au process is required (Fig. 4c). Once the well-defined Pt dual-rim NFs are prepared as shown in panel G, for Au dual-rim NFs, the intra-nanogaps can be controlled by changing the amount of Au ions during the regrowth of Au. For example, as we increase the amount of Au ions from 4 µl, 8 µl to 12 µl during the Au growth while keeping the other parameters the same, the distances of the intra-nanogaps decrease from 14, 8 to 5 nm and finally the gaps are merged if we further inject Au ions (Supplementary Fig. S10a–d). The corresponding optical profiles show continuous blueshifts of plasmonic bands from 720 to 692 to 686 nm with increased intensity due to the higher amount of Au as well as NFs with smaller gaps that seemingly behave like solid NPs (Supplementary Fig. 10e). It should be noted that all gap sizes reported in this work were determined from FE-SEM images.

### Single-particle SERS measurements

**Fig. 5:**
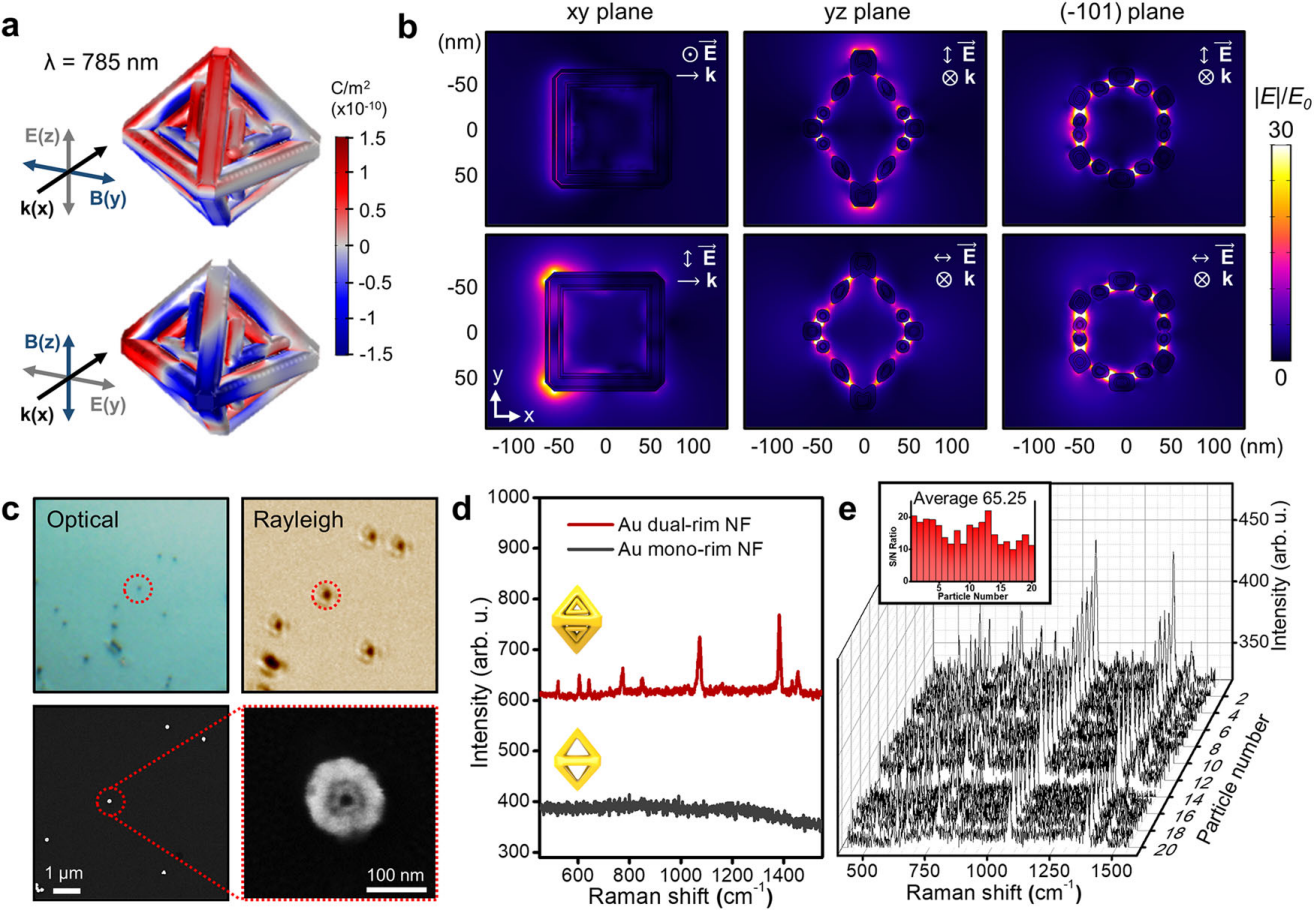
Optical characterization of Au octahedral dual-rim NFs. **a** Simulated surface charge density and **b** electromagnetic field distribution on Au octahedral dual-rim NFs under 785 nm excitation. The (−101) plane indicates the section of the diagonal face cut off from the Miller index of (−101) plane. **c** An optical microscopy image, corresponding Rayleigh scattering, and FE-SEM image of Au octahedral dual-rim NFs subjected to spSERS measurement. **d** Single-particle SERS spectra of Au octahedral mono- and dual-rim NFs. **e** Single-particle SERS spectra of 20 individual Au dual-rim NFs, demonstrating high reproducibility. The inset bar represents the signal-to-noise ratio of spSERS signals.

Highly concentrated electric fields, so-called hot-spots, can be produced via gaps or junctions at the nanometer scale between each metallic nanoparticle through interparticle, intraparticle plasmon coupling or sharp tips due to a well-known lightning-rod effect^28–31^. Therefore, strong and efficient light trapping of the electromagnetic field from octahedral dual-rim NFs is expected because of multiple narrow intra-nanogaps in a single 3D entity. To elucidate the nanogap-induced plasmonic coupling, we investigated the electromagnetic near-field distribution by a finite element method (FEM). Surface charge distribution mapping clearly exhibited that the surface charge is effectively separated at both the x- and y-polarized directions, and the electromagnetic near-field is highly focused at the narrow nanogaps between the dual rims under 785 nm excitation (Fig. 5a). In addition, due to the multiple resonant nanogaps produced by the dual frames, the near-field focusing is strongly enhanced between each rim and maximized (|E | /E_0_ = 933) at each vertex, which can be ascribed to the lightning-rod effect from the sharp tips under excitation of 785 nm (Fig. 5b). To experimentally verify the near-field focusing capability of dual-rim NFs, we performed a single-particle surface enhanced Raman scattering (spSERS) experiment using 2-naphthalenethiols as target probes. The NFs treated with Raman tags were randomly distributed on the transparent cover glass and the location of a single particle was confirmed by Rayleigh scattering and corresponding FE-SEM images (Fig. 5c and the experimental section provides further experimental details of spSERS). The spSERS spectra showed well-defined Raman spectral characteristics of the target analytes (concentration: 0.01 M, laser power: 170 µW) (Fig. 5d, red spectrum). This was mainly due to the multiple intra-nanogaps formed in a single entity, which is strongly responsible for electromagnetic field enhancement, enabling the acquisition of strong spSERS signals. Based on the spSERS measurements of at least 20 NFs, we confirmed that the spSERS signals were highly reproducible with strong Raman intensities, indicating highly effective near-field focusing capability and highly homogeneous size and shape of Au octahedral dual-rim NFs (Fig. 5e). We further tested Au octahedral mono-rim NFs for comparison, but these nanostructures did not show reliable spSERS signals (gray spectrum in Fig. 5d and see Supplementary Fig. 11 for an FE-SEM image and UV-vis spectra for the Au mono-rim NFs). The measured average enhancement factor (EF) of 3.68 × 10^7^ suggests that the Au octahedral dual-rim NFs can act as strong light entrappers (i.e., nano lenses). We recently reported that a larger particle size is one of the key strategies for achieving high EF due to increased Au mass^32^, thus we believe that there is more room to improve the EF of 3D Au dual-rim NFs with increasing the overall size, which is currently underway.

In addition to spSERS measurements, we conducted a liquid SERS measurement where Au dual-rim NFs were dispersed in solutions to eliminate substrate-induced effect on SERS signals and the clear SERS signals with high reproducibility were observed (Supplementary Fig. 12). In contrast, Au mono-rim NFs, Au octahedral solid NPs, 2D Au triangular dual-rim NFs (see a FE-SEM image in Supplementary Fig. 13) did not show any Raman features, indicating that the higher near-field collecting as well as increased number of hot spots of Au 3D dual-rim NFs is advantageous over other investigated analogous nanoparticles. Taken together, the increased number of hot-spot areas and the high surface areas (Au dual-rim NFs vs. Au mono-rim NFs) in 3D dual rim-engraved NFs enabled effective light confinement, which is supported by the spSERS and liquid SERS measurements. It should be noted that we have recently reported the synthesis of Au TOh dual-rim NFs where truncated flat terraces formed on six (100) facets facilitate the spontaneous formation of close-packed monolayer with no linker molecules^21^. Their bulk SERS substrates showed a highly sensitive detection capability because of a combinatorial effect from inter- and intra-particle coupling when they are assembled as a bulk substrate. However, polarization-sensitivity depending on the orientation of Au TOh dual-rim NFs hinders producing strong and uniform spSERS signals. When their dual-rim plane faced upwards, spSERS was observed, whereas no spSERS signals were observed when the flat terrace faced upwards because of no intra-particle coupling. In order to overcome these drawbacks, we designed Au octahedral dual-rim NFs, wherein all frames are faceted in an octahedral fashion, independent of the polarization directions of incoming light, which allows for uniform particle-by-particle spSERS signals (Fig. 5e) and hence effective nanoprobes for SERS-based biosensing immunoassay (*vide infra*).

### SERS-based immunoassay toward human chorionic gonadotropin (HCG)

**Fig. 6:**
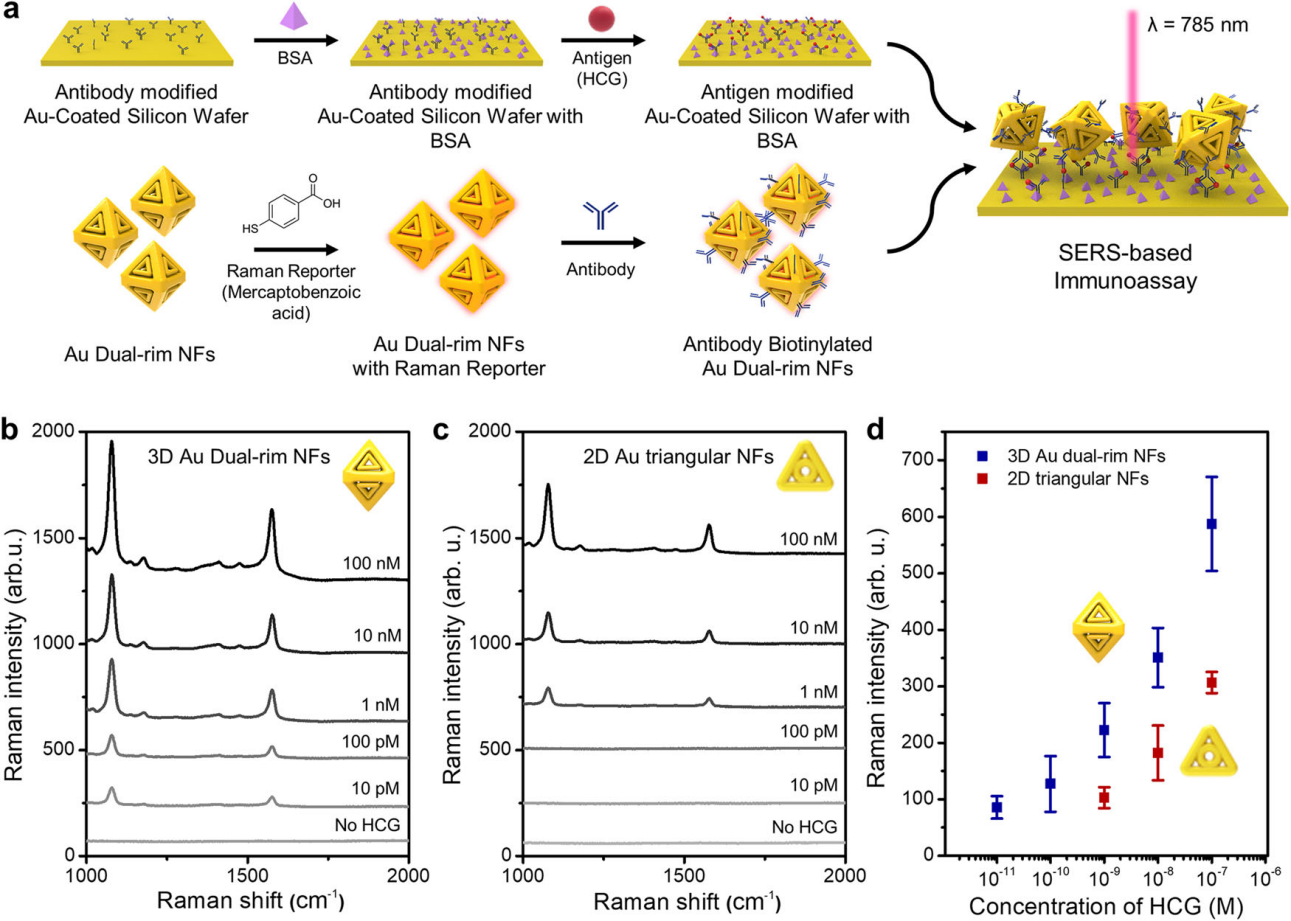
SERS-based Immunoassay of Au dual-rim NFs toward detection of HCG. **a** A schematic illustration of SERS-based immunoassay of 3D Au dual-rim NFs toward detection of HCG. Raman spectra achieved using **b** 3D Au dual-rim NFs and **c** 2D Au triangular dual-rim NFs with different concentrations of HCG and **d** corresponding calibration curves for the limit of detection of each NF. The blue points represent 3D Au dual-rim NFs, and the red points represent 2D Au triangular NFs. The error bars represent standard deviations from *n* = 3. The experimental details and characterization information for 2D Au triangular NFs can be found in ref. 33.

To provide the applicability of 3D Au dual-rim NFs, we conducted a SERS-based immunoassay toward human chorionic gonadotropin (HCG, a hormone found in women’s body during pregnancy), exploiting a sandwich platform as described in the schematic description in Fig. 6a (see Methods section for detailed experiments). For comparison, 2D Au triangular dual-rim NFs were synthesized by following our previous protocol^33^ and the size (97 ± 4 nm) was chosen to be comparable to the outer diameter of 3D Au dual-rim NFs (93 ± 3 nm). SERS spectra achieved from 3D Au dual-rim NFs and 2D Au triangular dual-rim NFs with different HCG concentrations are represented in Fig. 6b, c respectively. It is clearly observed that the Raman intensities from 3D Au dual-rim NFs are higher than those from 2D Au triangular NFs over all investigated ranges of HCG concentrations. In addition, when we plotted the calibration curves for both NFs (Fig. 6d), the limit of detection (LOD) is found to be 10 pM in the case of 3D Au dual-rim NFs (blue dots), two-order lower than that of 2D triangular dual-rim NFs (1 nM) (red dots), clearly demonstrating that strong near-field focusing of 3D Au dual-rim NFs due to eight intra-nanogaps as well as increased probability of analytes trapping on hot spots.

In conclusion, we have shown the synthetic method of complex NF structures where dual rims are formed and engraved on each facet of octahedral NFs by adopting multi-stepwise chemical reactions. The well-faceted growth step is the key to the successful synthesis of dual-rim NFs wherein Au grows following a layer-by-layer growth mode and generates sharp edges, facilitating Pt deposition on the inner and outer Au mono-rim NFs. The synthesis scheme described herein enabled the realization of complex plasmonic dual-rim NFs with multiple resonant intra-nanogaps between inner and outer rims, which enhanced the electric near-field confinement, as further verified by single-particle SERS analysis. In addition, the strong near-field focusing capability of 3D dual-rim NFs in a single entity was theoretically supported by the surface charge density distribution and electromagnetic field distribution calculations. Importantly, we applied 3D Au dual-rim NFs to SERS-based immunoassay and showed the potential usefulness as nanoprobes for biosensing. We expect that this colloidal chemistry for 3D dual-rim NFs will contribute to designing more complex nanoparticles with advanced optical and physicochemical properties.

## Methods

### Au octahedral nanoparticles (NPs)

The Au octahedral nanoparticles were prepared by a seed-mediated three-step growth process^11^. First, to synthesize the Au seed, 7 mL of 75 mM CTAB, 87.5 μL of 20 mM HAuCl_4_, and 600 μL of 10 mM NaBH_4_ (ice-cold (15 min)) were added to a vial and stirred at 700 rpm for 3 h. In the first growth process, 480 mL of 16 mM CTAB, 200 μL of 20 mM HAuCl_4_, and 6 mL of 0.1 M ascorbic acid (A.A) were mixed in a round volumetric flask and 6 mL of Au seed was added (100x dilution). The mixture was kept at 30 °C for `12 h. In the second growth process, 210.8 ml of 100 mM CTAB, 1.4 mL of 20 mM HAuCl_4_, and 6.53 ml of 0.1 M A.A were mixed in a round volumetric flask and added to 140.5 mL of the 1st growth Au octahedral NPs. The mixture was kept at 30 °C for ~4 h. In the third growth process, 320.7 ml of 100 mM CTAB, 2.1 mL of 20 mM HAuCl_4_, and 9.9 ml of 0.1 M A.A were mixed in a round volumetric flask and added to 134.7 mL of the 2nd growth Au octahedral. The mixture was kept at 30 °C for ~4 h.

### Au@Pt octahedral NPs

To synthesize Au@Pt octahedral NPs, 15 mL of 50 mM CTAB, 5 mL of the 3rd growth of Au octahedral NPs, 10 μL of 0.2 mM AgNO_3_, and 600 μL of 0.1 M A.A were added to a vial in the presence of iodide ions (50 μM). The solution was heated to 70 °C and kept in an oven to promote the deposition of Ag layers onto the Au octahedral NPs. After 1 h, 600 μL of 0.1 M HCl and 100 μL of a 2 mM aqueous H_2_PtCl_6_ solution were injected into the growth solution. The mixture was kept at 70 °C for ~2.5 h. After completion of the reaction, we washed the samples in a centrifuge at 3,663 g for 20 min and repeated the process three times.

### Synthesis of Pt octahedral mono-rim nanoframes (NFs)

To prepare the Pt octahedral mono-rim NFs, 15 mL of 50 mM CTAB, 400 μL of 0.2 mM HAuCl_4_, and 10 mL of Au@Pt octahedral NPs were combined in the presence of iodide ions (50 μM). The etching step took 45 min at 50 °C. After completion of the reaction, we washed the samples in a centrifuge at 9,358 g for 10 min.

### Well-faceted growth of Au on the Pt mono-rim NFs

Au mono-rim NFs with sharp edges were synthesized through a well-faceted growth step by reducing Au ions in the presence of chloride. First, 250 μL of Pt mono-rim NF dispersed in 0.2 M CTAC, 13 μL of 2 mM AgNO_3_, and 13 μL of 2 mM HAuCl_4_ were added to a 1.5 ml Eppendorf tube. Then, 9 μL of 0.01 M A.A was added. The reaction solution was kept at 50 °C in an oven for 30 min. Residual Au ions were washed by centrifugation at 9,358 g for 5 min.

### Synthesis of Au@Pt octahedral NFs

In the presence of iodide ions (50 μM), 15 mL of 0.05 M CTAB, 5 mL of redispersed inwardly grown Au frame nanoparticles of the well-faceted growth mode, 1 ml of 50 mM CTAB, 250 μL of well-faceted Au NFs, 0.85 μL of 0.2 mM AgNO_3_, and 30 μL of 0.1 M ascorbic acid were added to a vial. The solution was heated to 70 °C and kept in an oven to promote the deposition of Ag layers onto the Au octahedral NFs. After 1 h, 30 μL of 0.1 M HCl and 8.5 μL of a 2 mM aqueous H_2_PtCl_6_ solution were added into the growth solution. The mixture was kept at 70 °C for ~2.5 h. After completion of the reaction, we washed the samples in a centrifuge at 3663 g for 20 min and repeated this step three times.

### Synthesis of Pt octahedral dual-rim NFs

1 mL of 50 mM CTAB, 18 μL of 0.2 mM HAuCl_4_, and 250 mL of Au@Pt octahedral NPs were combined in the presence of iodide ions (50 μM). The etching process took 45 min at 50 °C. After completion of the reaction, we washed the samples in a centrifuge at 9,358 g for 10 min.

### Synthesis of Au octahedral dual-rim NFs

250 μL of dual Pt NF dispersed in 0.2 M CTAC, 9 μL of 2 mM AgNO_3_, and 9 μL of 2 mM HAuCl_4_ were added to a 1.5 ml Eppendorf tube. Then, 9 μL of 0.01 M AA was added. The reaction solution was kept at 50 °C in an oven for 30 min. Residual gold ions were removed by means of centrifugation at 9,358 g for 5 min.

### Characterization

Field-emission scanning electron microscopy (FE-SEM) images were obtained using JSM-7100F and JSM-7800F instruments (JEOL) at the Chiral Material Core Facility Center of Sungkyunkwan University. JEM-2100F and JEM-ARM 200 F instruments (JEOL) were used to acquire transmission electron microscopy (TEM) images. UV-vis-NIR absorption spectra were acquired using a spectrophotometer (Shimadzu UV-3600). 3D tomography images were obtained using a Talos F200X. An NX10 instrument (Park systems) was used to acquire AFM images and height profiles.

### Single-particle SERS (spSERS) measurement

The spSERS measurements were conducted using a Raman microscope (Ntegra, NT-MDT) equipped with an inverted optical microscope (IX 73, Olympus). A dichroic mirror directs the excitation laser beam into the oil immersion objective (UPlanSApo, 100×, 1.4 numerical aperture), which focuses the beam to a diffraction-limited spot (~2 μm) on the upper surface of the cover glass slip. Photomultiplier tube images were obtained using a piezoelectric x, y sample scanner to identify nanoparticles. The SERS spectra were acquired with a 785 nm laser (170 μW) for 10 s. The signals were detected by a charge-coupled device detector (1024 × 256 pixels; Peltier cooled to - 70 °C, Andor Newton DU920P BEX2-DD). After analysis, FE-SEM images of the samples were obtained after Pt layer deposition using an Ar plasma sputter-coater (Cressington 108 auto) with a current level of 20 mA for 60 s on the slide glass.

### Calculation of the enhancement factor

The enhancement factor of Raman signals was based on the following Eq. (1):

1 $${{{{{\rm{EF}}}}}}=\frac{{I}_{{{{{{\rm{SERS}}}}}}}}{{I}_{{{{{{\rm{bulk}}}}}}}}\times \frac{{N}_{{{{{{\rm{bulk}}}}}}}}{{N}_{{{{{{\rm{SERS}}}}}}}}$$

*I*_SERS_ and *I*_bulk_ are the Raman intensities of 2-naphthalenethiol (2-NTT) for surface adsorption and solution samples, respectively. *N*_bulk_ is the number of 2-NTT molecules in the volume for the normal Raman signals, and N_SERS_ is the number of 2-NTT absorbed on the Au octahedral dual-rim nanoframes for the SERS signal. The SERS peak, which is a ring stretching mode at ~1380 cm^−1^, was chosen for the EF calculation. *N*_bulk_ was calculated using the following Eq. (2):

2 $${N}_{{{{{{\rm{bulk}}}}}}}=\left(\frac{V\times d}{{M}_{{{{{{\rm{W}}}}}}}}\right)\times {N}_{{{{{{\rm{A}}}}}}}=\left(\frac{{{{{{\rm{\pi }}}}}}\times {r}^{2}\times h\times d}{{M}_{{{{{{\rm{W}}}}}}}}\right)\times {N}_{{{{{{\rm{A}}}}}}}=2.67\times {10}^{12}$$

where *V* is the excitation volume of the 2-NTT substrate, *d* is the density of 2-NTT (1.2 g/cm^3^), *M*_W_ is the molecular weight of 2-NTT (160.23 g/mol), *N*_A_ is Avogadro’s number (6.02 × 10^23 ^mol^−1^), *r* is the radius of the laser beam (~2 μm), and *h* is the focal depth of the laser (~47 μm).

*N*_SERS_ was calculated by using the following Eq. (3):

3 $${N}_{{{{{{\rm{SERS}}}}}}}=A\times D\times {N}_{{{{{{\rm{A}}}}}}}=8.80\times {10}^{4}$$

where *A* is the SERS-active area of the 3D Au octahedral dual-rim nanoframes with inner and outer rings (3.54 × 10^−10^ cm^2^), which was measured as entire surface area of the 3D Au octahedral dual-rim nanoframes. *D* is an estimated density of 2-NTT molecules in single-assembled monolayer on Au (4.0 × 10^−10 ^mol/cm^2^).

### Surface modification of NPs with detection antibodies

We modified the NPs with detection antibodies (HCG recombinant rabbit monoclonal antibody (RM330), purchased from Sigma-Aldrich) for SERS-based immunoassay by decorating the surface of NPs with 4-mercaptobenzoic acids (4-MBA). We mixed 500 μL of NPs with 100 μL of 1 mM 4-MBA (dissolved in 10% ethanol solution). After the reaction at 70 °C for 30 min, we washed the resulting solution with centrifugation at 13,475 g for 5 min to remove residual 4-MBA molecules. To immobilize detection antibodies at the surface of NPs, we conducted EDC/NHS coupling to activate the carboxylic acid functional group of 4-MBA immobilized at the surface of NPs. We added 100 μL of EDC/NHS solution (80 mM of N-hydroxysuccinimide and 40 mM of N-(3-dimethylaminopropyl)-N’-ethylcarbodiimide dissolved in MES buffer (pH 6.5) with TWEEN^®^ 20 (0.5%)) solution to the 100 μL of NP solution. After the reaction at room temperature for 10 min, we washed the resulting solution with centrifugation at 13,475 g for 5 min to remove residual EDC/NHS solution. To immobilize antibodies at the surface of NPs, we introduced 100 μL of antibodies (HCG recombinant rabbit monoclonal antibody, 1:10000 dilution in PBS) to the carboxylic acid group activated NPs. After the reaction at room temperature for 10 min, we washed the resulting solution with centrifugation at 13,475 g for 5 min to remove residual antibodies and dispersed in PBS.

### Surface modification of Au-coated silicon wafer substrates with capture antibodies for immunoassay

We modified the Au-coated silicon wafers (4 mm × 4 mm, the thickness of Ti layer: 100 Å, the thickness of Au layer: 1000 Å) with capture antibodies for SERS-based immunoassay. To decorate the Au-coated silicon wafer, we washed the surface of Au-coated silicon wafer through sonication in ethanol for 10 min. Then, we immersed Au-coated silicon wafer in the 0.1 M 3-mercaptopropionic acid (3-MPA) solution to generate carboxylic acid functional groups at the surface of Au-coated silicon wafers. After the reaction at room temperature for 6 h, we washed the surface with ethanol to remove residual 3-MPA molecules. To activate carboxylic acid groups for the formation of amide bonding with capture antibodies, we conducted EDC/NHS coupling. We immersed 3-MPA decorated Au-coated silicon wafer in the 20 mL of EDC/NHS solution (80 mM of N-hydroxysuccinimide and 40 mM of N-(3-dimethylaminopropyl)-N’-ethylcarbodiimide dissolved in MES buffer (pH 6.5) with TWEEN 20 (0.5%)) for 30 min. After EDC/NHS coupling, we washed Au-coated silicon wafer with PBS. To immobilize the capture antibodies on the surface of Au-coated silicon wafer, we dropped 4 μL of antibodies (Anti-hCG antibody, mouse polyclonal antibody, 1:1000 dilution in PBS) on the carboxylic acid group activated Au-coated silicon wafer. After the reaction at room temperature for 30 min, we dropcasted 4 μL of 5% bovine serum albumin (BSA) solution on the Au-coated silicon wafer.

### SERS-based immunoassay toward HCG

We introduced 4 μL of HCG hormone (diluted with PBS) with varying concentration on the capture antibody-modified Au-coated silicon wafer, which was incubated at room temperature for 10 min for immunochemical reaction between antigens and capture antibodies modified on the Au-coated silicon wafer. After the incubation, we injected 4 μL of detection antibody-modified nanoparticles on the antigen-antibody modified Au-coated silicon wafer, which was incubated at room temperature for 5 min for immunochemical reaction between antigens immobilized on the Au-coated silicon wafer and detection antibodies modified at the NPs. After 5 min, we washed the Au-coated silicon wafer with PBS. After washing, we removed moisture on the silicon wafer with N_2_ gas. Then, we conducted SERS mapping measurements for immunoassay (mapping area: 200 μm × 200 μm, with 20 μm interval). We monitored the peak at 1080 cm^−1^ (aromatic ring vibration mode of 4-MBA) with a 785 nm laser excitation (laser power: 4 mW) with an integration time of 500 ms.

### Reporting summary

Further information on research design is available in the Nature Research Reporting Summary linked to this article.

## Supplementary information

Supplementary Information

Description of Additional Supplementary Files

Supplementary Movie 1

Reporting Summary

## Data Availability

The data that support the findings of this study are available from the corresponding authors upon request.
