# Supplementary material for: Three-dimensional nanoframes with dual rims as nanoprobes for biosensing
Source: Nat Commun. 2022 Aug 16;13:4813. doi: 10.1038/s41467-022-32549-w (PMC9381508; doi:10.1038/s41467-022-32549-w)
Supplement: Supplementary file 1 — Supplementary Information [file 41467_2022_32549_MOESM1_ESM.pdf]

Supplementary Information

Three-dimensional nanoframes with dual rims as nanoprobes for biosensing

Hajir Hilal[a†], Qiang Zhao[a†], Jeongwon Kim[a], Sungwoo Lee[a], MohammadNavid Haddadnezhad[a], Sungjae Yoo[a], Soohyun Lee[a], Woongkyu Park[b], Woocheol Park[a], Jaewon Lee[a], Joong Wook Lee[c], Insub Jung[a,d]* and Sungho Park[a]*

[a]Department of Chemistry, Sungkyunkwan University (SKKU), Suwon 16419, Republic of Korea

[b]Medical & Bio Photonics Research Center, Korea Photonics Technology Institute (KOPTI), Gwangju, 61007, Republic of Korea

[c]Department of Physics and Optoelectronics Convergence Research Center, Chonnam National University, Gwangju 61186, Republic of Korea

[d]Department of Chemistry and Institute of Basic Science, Sungkyunkwan University (SKKU), Suwon 16419, Republic of Korea

e-mail: insub.jung@skku.edu; spark72@skku.edu

†: these authors contributed equally

Table of contents

Supplementary Figure 1. Selective Pt deposition and etching of inner Au. FE-SEM images of **(a)** Au octahedral NPs, **(b)** Au@Pt NPs, and **(c)** Pt octahedral mono-rim NFs. **(d)** Corresponding UV-Vis-NIR spectra. 4

Supplementary Figure 2. The effect of Ag precursors during the well-faceted growth of Au octahedral mono-rim NFs. (a-c) FE-SEM images of Au octahedral mono-rim NFs with different gap distances as the amount of Au increases under the presence of Ag ions (well-faceted growth) and **(d)** corresponding UV-vis-NIR spectra with sequential blue-shifts of plasmonic bands from 854 to 762 to 700 nm. **(e-g)** FE-SEM images of Au octahedral tip-blobbed NFs with increasing Au ions without Ag ions and **(f)** corresponding UV-vis-NIR spectra with plasmonic blue-shifting from 870 to 724 to 636 nm. 5

Supplementary Figure 3. Growth pattern control of Au on the 3D PtAu skeleton. (a) FE-SEM images of different Au growth patterns on the 3D PtAu skeletons with different halide ions and pH of the reaction solution under the presence of Ag^{+}. **(b)** Summarized experimental conditions for growth pattern control of Au. Depending on counter anions, Au mono-rim NFs with rough surface, smooth surface, or well-faceted sharp surfaces. 6

Supplementary Figure 4. Controlling the amount of the Ag^{+} with regard to Au^{3+} during the reactions. FE-SEM images of Au mono-rim NFs with different $[Ag^{+}]/[Au^{3+}]$ ratios of **(a)** 0.05 **(b)** 0.1 **(c)** 1. A red box indicates the successful case of well-faceted growth of Au mono-rim NFs. All scale bars = 100 nm. 7

Supplementary Figure 5. Growth evolution of Pt dual-rim NFs starting from Au mono-rim NFs with a round tip or smooth surfaces. FE-SEM images of **(a)** Au round-tip NFs, **(b)** after the second Pt rim-on deposition, **(c)** Pt dual-rim NFs after the selective inner Au etching, and **(d)** UV-vis-NIR spectra that correspond to panel **a** (black line), **b** (red line) and **c** (blue line). **(e)** When we use Au mono-rim NFs with smooth surfaces as initial templates, **(f)** Pt is not preferentially deposited along the edges, leading to **(g)** dull-like Pt dual-rim NFs. **(h)** UV-vis-NIR spectra that correspond to **e** (black line), **f** (red line) and **g** (blue line). 8

Supplementary Figure 6. HADDF-STEM EDS elemental analysis of Au dual-rim NFs. The spectrum reveals the compositions (Au, Ag, Pt) of Au octahedral dual-rim NFs. The atomic percentage of Au octahedral dual-rim NFs are Ag (10.04 %), Au (75.79 %), and Pt (14.18 %), respectively. 9

Supplementary Figure 7. Calculated optical extinction of Au dual-rim NFs under an electric field with different oscillating field directions. Simulated absorption, scattering, and extinction spectra under an electric field of light oscillating along the **(a)** z-axis and **(b)** y-axis. 10

Supplementary Figure 8. Dark-field spectra of Au dual-rim NFs. We obtained dark-field scattering spectra of individual single Au dual-rim NFs and observed that the scattering bands were centered around 695 nm to 720 nm. Inset shows an optical image and each number indicate single Au dual-rim NFs. The scale bar in the inset denotes 1 μm. 11

Supplementary Figure 9. Control of the gap distances between the inner and outer rims of Pt octahedral dual-rim NFs. (a, d, g) Well-faceted Au octahedral mono-rim NFs with different rim thicknesses. **(b, e, h)** Pt@Au@Pt octahedral NFs after selective Pt deposition along the edges of Au mono-rim NFs. **(c)** Corresponding UV-vis-NIR spectra. Black and red lines correspond to panel **a** and **b**, respectively. A blue line corresponds to Fig. 2h in the main text. **(f)** Corresponding UV-vis-NIR spectra. Black and red lines correspond to panel **d** and **e**, respectively. A blue line corresponds to Fig. 2g in the

main text. **(i)** Corresponding UV-vis-NIR spectra. Black and red lines correspond to panel **g** and **h**, respectively. A blue line corresponds to Fig. 2f in the main text. All scale bars = 100 nm. 12

Supplementary Figure 10. Control of the gap distances between the inner and outer rims in Au octahedral dual-rim NFs. (a) Pt dual-rim NFs with a gap size of 17 nm. **(b-d)** Au octahedral dual-rim NFs with different gap distances from 14 nm to 5 nm, controlled by the amount of Au^{3+} added to the reaction solutions. The cartoons represent the size information of each nanostructure, and the numbers denote the sizes in nm. **(e)** UV-vis-NIR spectra of Au dual-rim NFs with different gap sizes. The error bars represent standard deviations from 30 particles. 13

Supplementary Figure 11. An FE-SEM image and UV-vis spectrum for the Au mono-rim NFs. (a) An UV-vis-NIR spectrum and **(b)** an FE-SEM image of Au octahedral mono-rim NFs. **(c, d)** Single-particle SERS measurements of 10 individual Au octahedral mono-rim NFs. 14

Supplementary Figure 12. Liquid SERS measurements. (a) A schematic illustration of liquid SERS measurements. **(b)** Raman spectra of Au dual-rim NFs toward 2-NTT in solution phase. Only 3D Au dual-rim NFs show Raman signals of 2-NTT. **(c)** SERS spectra of Au dual-rim NFs dispersed in solution (n = 30), demonstrating high reproducibility. 15

Supplementary Figure 13. An FE-SEM image of 2D Au triangular dual-rim NFs. The size of 2D Au triangular dual-rim NFs (97±4 nm) is selected to be comparable to the outer diameter of 3D Au dual-rim NFs (93±3 nm). The synthesis method for 2D Au triangular dual-rim NFs can be found in the previous literature.[1] 16

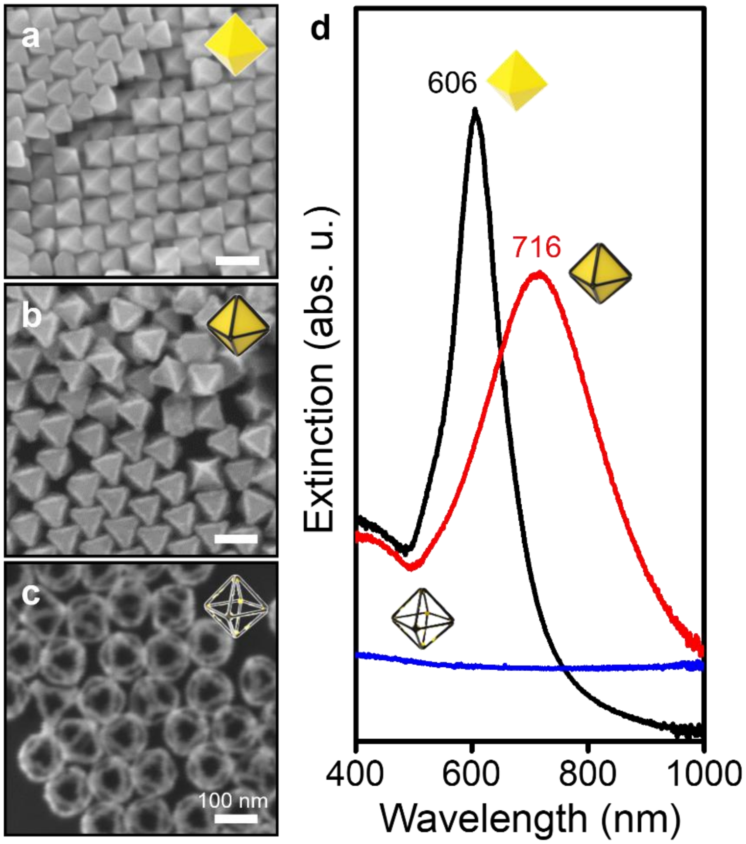

Supplementary Figure 1. Selective Pt deposition and etching of inner Au. FE-SEM images of **(a)** Au octahedral NPs, **(b)** Au@Pt NPs, and **(c)** Pt octahedral mono-rim NFs. **(d)** Corresponding UV-Vis-NIR spectra.

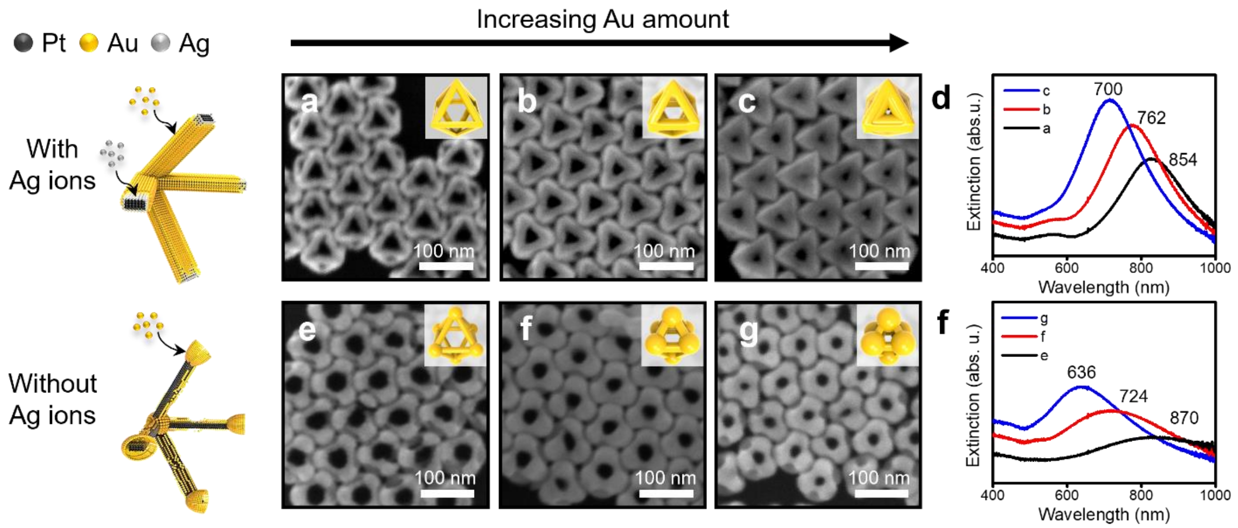

Supplementary Figure 2. The effect of Ag precursors during the well-faceted growth of Au octahedral mono-rim NFs. (a-c) FE-SEM images of Au octahedral mono-rim NFs with different gap distances as the amount of Au increases under the presence of Ag ions (well-faceted growth) and **(d)** corresponding UV-vis-NIR spectra with sequential blue-shifts of plasmonic bands from 854 to 762 to 700 nm. **(e-g)** FE-SEM images of Au octahedral tip-blobbed NFs with increasing Au ions without Ag ions and **(f)** corresponding UV-vis-NIR spectra with plasmonic blue-shifting from 870 to 724 to 636 nm.

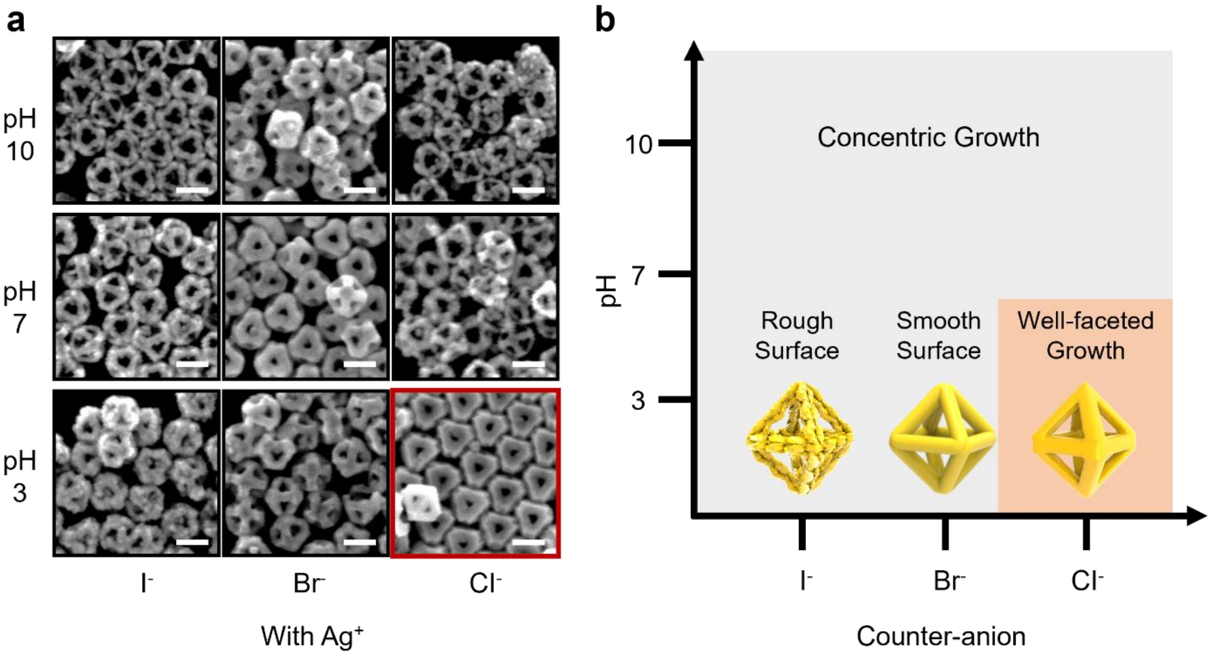

Supplementary Figure 3. Growth pattern control of Au on the 3D PtAu skeleton. (a) FE-SEM images of different Au growth patterns on the 3D PtAu skeletons with different halide ions and pH of the reaction solution under the presence of Ag^+. **(b)** Summarized experimental conditions for growth pattern control of Au. Depending on counter anions, Au mono-rim NFs with rough surface, smooth surface, or well-faceted sharp surfaces.

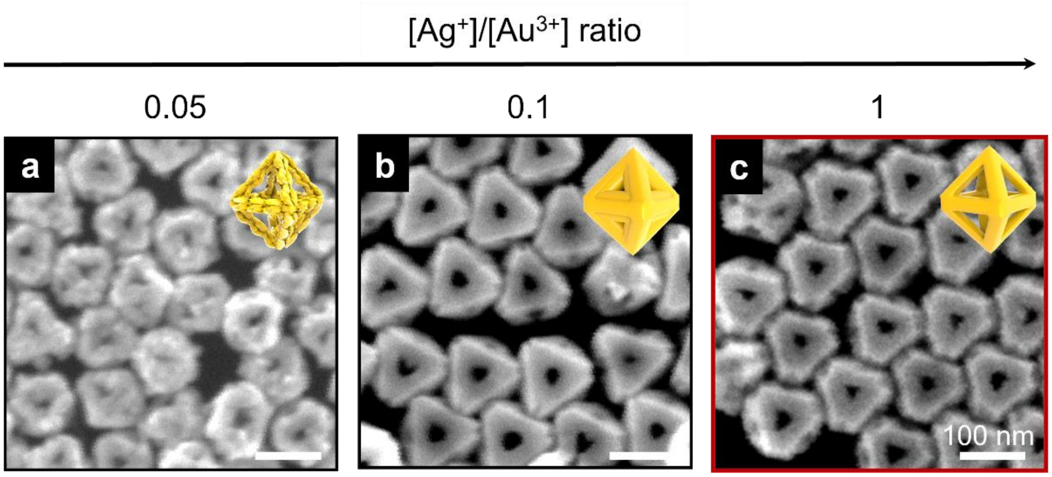

Supplementary Figure 4. Controlling the amount of the Ag^{+} with regard to Au^{3+} during the reactions. FE-SEM images of Au mono-rim NFs with different $[Ag^{+}]/[Au^{3+}]$ ratios of **(a)** 0.05 **(b)** 0.1 **(c)** 1. A red box indicates the successful case of well-faceted growth of Au mono-rim NFs. All scale bars = 100 nm.

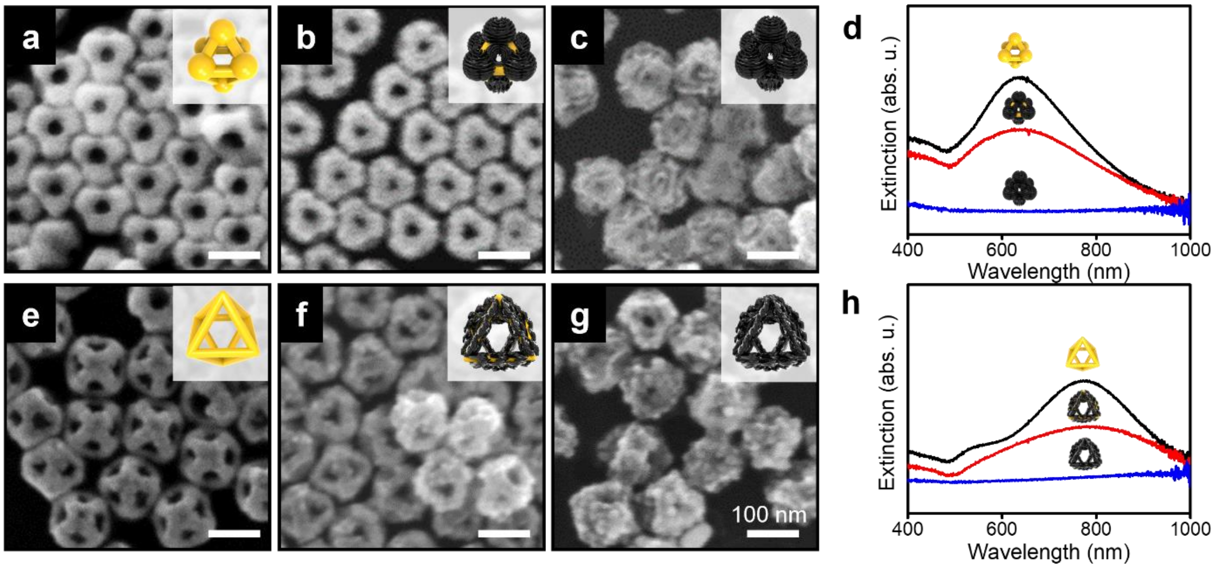

Supplementary Figure 5. Growth evolution of Pt dual-rim NFs starting from Au mono-rim NFs with a round tip or smooth surfaces. FE-SEM images of **(a)** Au round-tip NFs, **(b)** after the second Pt rim-on deposition, **(c)** Pt dual-rim NFs after the selective inner Au etching, and **(d)** UV-vis-NIR spectra that correspond to panel **a** (black line), **b** (red line) and **c** (blue line). **(e)** When we use Au mono-rim NFs with smooth surfaces as initial templates, **(f)** Pt is not preferentially deposited along the edges, leading to **(g)** dull-like Pt dual-rim NFs. **(h)** UV-vis-NIR spectra that correspond to **e** (black line), **f** (red line) and **g** (blue line).

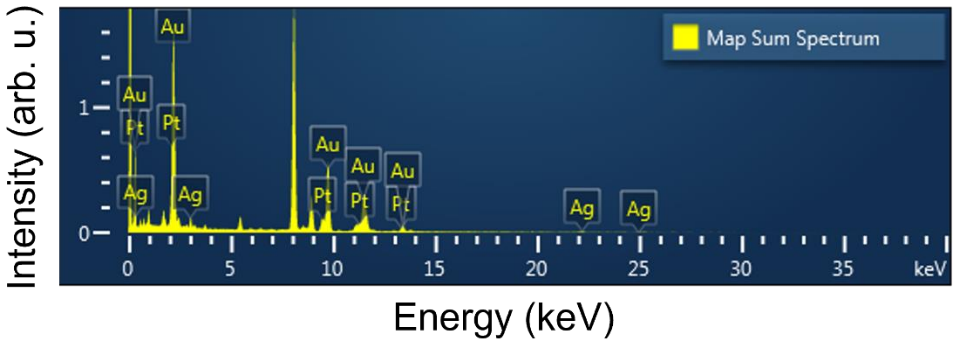

Supplementary Figure 6. HADDF-STEM EDS elemental analysis of Au dual-rim NFs. The spectrum reveals the compositions (Au, Ag, Pt) of Au octahedral dual-rim NFs. The atomic percentage of Au octahedral dual-rim NFs are Ag (10.04 %), Au (75.79 %), and Pt (14.18 %), respectively.

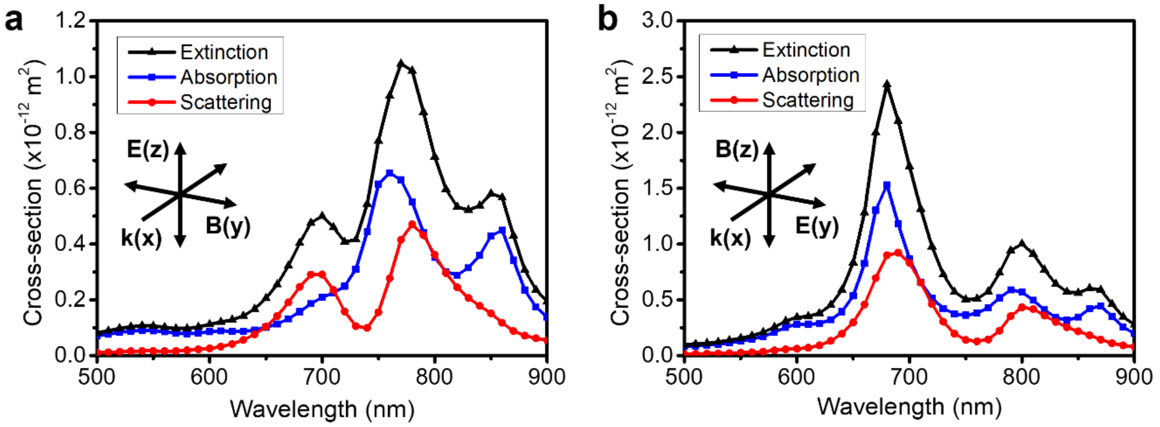

Supplementary Figure 7. Calculated optical extinction of Au dual-rim NFs under an electric field with different oscillating field directions. Simulated absorption, scattering, and extinction spectra under an electric field of light oscillating along the **(a)** z-axis and **(b)** y-axis.

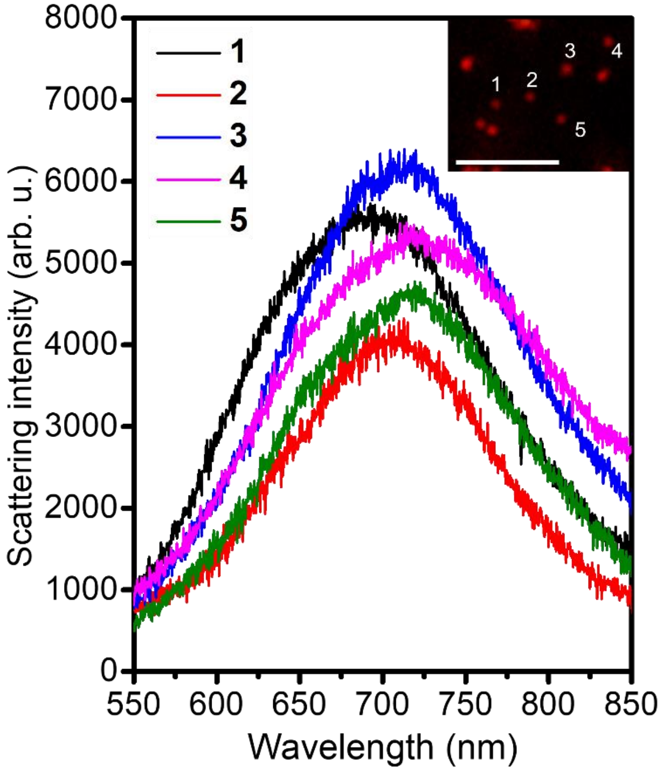

Supplementary Figure 8. Dark-field spectra of Au dual-rim NFs. We obtained dark-field scattering spectra of individual single Au dual-rim NFs and observed that the scattering bands were centered around 695 nm to 720 nm. Inset shows an optical image and each number indicate single Au dual-rim NFs. The scale bar in the inset denotes 1 μm.

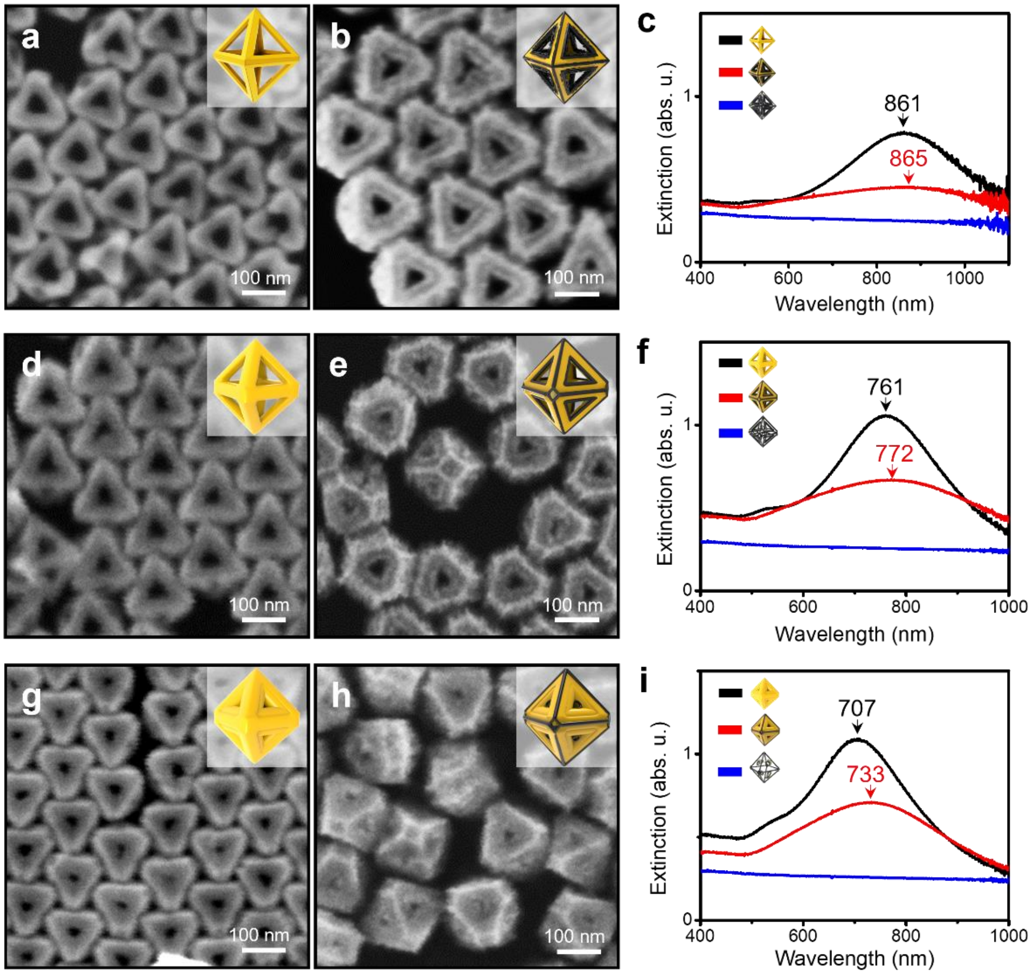

Supplementary Figure 9. Control of the gap distances between the inner and outer rims of Pt octahedral dual-rim NFs. (a, d, g) Well-faceted Au octahedral mono-rim NFs with different rim thicknesses. **(b, e, h)** Pt@Au@Pt octahedral NFs after selective Pt deposition along the edges of Au mono-rim NFs. **(c)** Corresponding UV-vis-NIR spectra. Black and red lines correspond to panel **a** and **b**, respectively. A blue line corresponds to Fig. 2h in the main text. **(f)** Corresponding UV-vis-NIR spectra. Black and red lines correspond to panel **d** and **e**, respectively. A blue line corresponds to Fig. 2g in the main text. **(i)** Corresponding UV-vis-NIR spectra. Black and red lines correspond to panel **g** and **h**, respectively. A blue line corresponds to Fig. 2f in the main text. All scale bars = 100 nm.

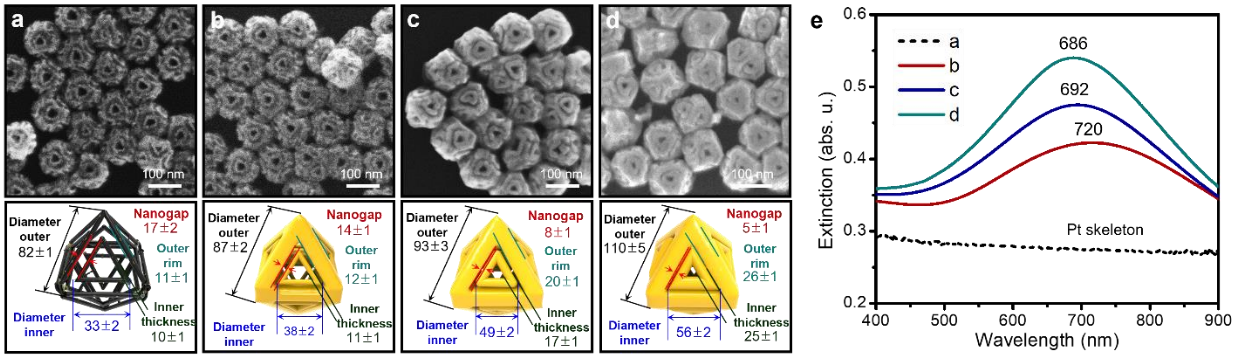

Supplementary Figure 10. Control of the gap distances between the inner and outer rims in Au octahedral dual-rim NFs. (a) Pt dual-rim NFs with a gap size of 17 nm. **(b-d)** Au octahedral dual-rim NFs with different gap distances from 14 nm to 5 nm, controlled by the amount of Au^{3+} added to the reaction solutions. The cartoons represent the size information of each nanostructure, and the numbers denote the sizes in nm. **(e)** UV-vis-NIR spectra of Au dual-rim NFs with different gap sizes. The error bars represent standard deviations from 30 particles.

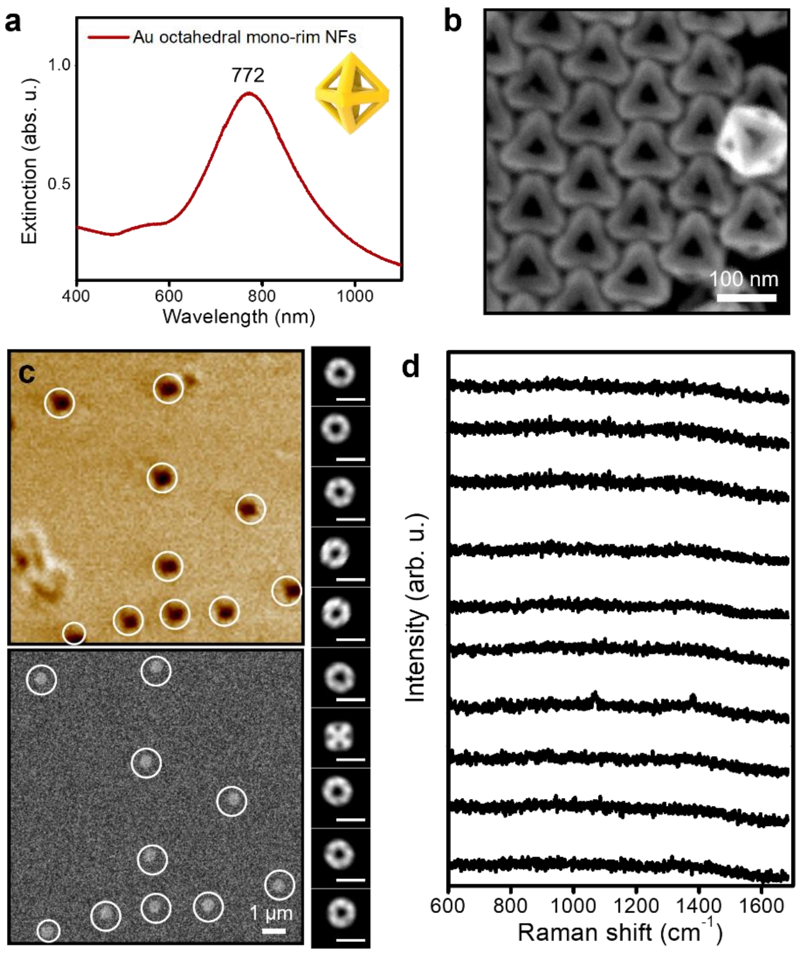

Supplementary Figure 11. An FE-SEM image and UV-vis spectrum for the Au mono-rim NFs. **(a)** An UV-vis-NIR spectrum and **(b)** an FE-SEM image of Au octahedral mono-rim NFs. **(c, d)** Single-particle SERS measurements of 10 individual Au octahedral mono-rim NFs.

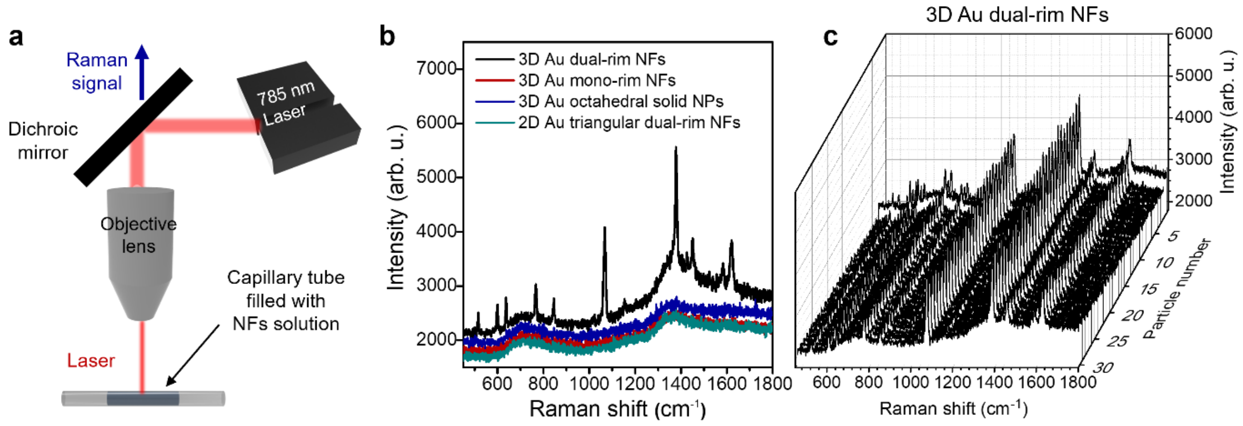

Supplementary Figure 12. Liquid SERS measurements. (a) A schematic illustration of liquid SERS measurements. **(b)** Raman spectra of Au dual-rim NFs toward 2-NTT in solution phase. Only 3D Au dual-rim NFs show Raman signals of 2-NTT. **(c)** SERS spectra of Au dual-rim NFs dispersed in solution (n = 30), demonstrating high reproducibility.

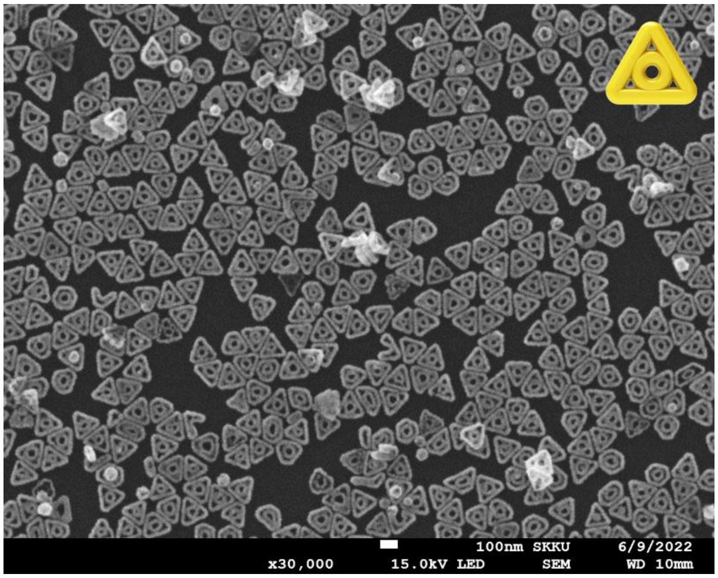

Supplementary Figure 13. An FE-SEM image of 2D Au triangular dual-rim NFs. The size of 2D Au triangular dual-rim NFs (97±4 nm) is selected to be comparable to the outer diameter of 3D Au dual-rim NFs (93±3 nm). The synthesis method for 2D Au triangular dual-rim NFs can be found in the previous literature.[1]

Supplementary References

1. Go S*, et al.* Ring-in-a-Triangle Nanoframes: Integrating with Intra- and Interhotspots for Highly Amplified Near-Field Focusing. *Nano Letters* **22**, 1734-1740 (2022).
