# Supplementary material for: Three-dimensional nanoframes with dual rims as nanoprobes for biosensing
Source: Nat Commun. 2022 Aug 16;13:4813. doi: 10.1038/s41467-022-32549-w (PMC9381508; doi:10.1038/s41467-022-32549-w)
Supplement: Supplementary file 2 — Description of Additional Supplementary Files [file 41467_2022_32549_MOESM2_ESM.pdf]

<u>Description of additional supplementary files:</u>

Supplementary Movie 1 – A 3D tomographic video of 3D Au dual-rim NFs.
